# Molecular detection and typing of pathogenic *Leptospira* species from livestock and small mammals in Uganda

**DOI:** 10.1017/S0950268825000044

**Published:** 2025-01-22

**Authors:** Lordrick Alinaitwe, Martin Wainaina, Salome Dürr, Clovice Kankya, Velma Kivali, James Bugeza, Christopher Joshua Aturinda, Ashiraf Lubega, Anne Mayer-Scholl, Jolly Justine Hoona, Peter Bahn, Jens Andre Hammerl, Kristina Roesel, Elizabeth Anne Jessie Cook, Martin H. Richter

**Affiliations:** 1 Human and Animal Health Program, International Livestock Research Institute, Nairobi, Kenya; 2 Veterinary Public Health Institute, University of Bern, Bern, Switzerland; 3 Graduate School for Cellular and Biomedical Sciences, University of Bern, Bern, Switzerland; 4 College of Veterinary Medicine, Animal Resources and Biosecurity (COVAB), Makerere University, Kampala, Uganda; 5 Department of Biological Safety, German Federal Institute for Risk Assessment, Berlin, Germany; 6 Vaccinology Research Program, National Livestock Resources Research Institute, Kampala, Uganda; 7 Department of Animal Production, Ministry of Agriculture, Animal Industry and Fisheries (MAAIF), Entebbe, Uganda; 8 Hans-Ruthenberg-Institute for Tropical Agricultural Sciences, University of Hohenheim

**Keywords:** leptospirosis, molecular epidemiology, livestock, Uganda, rodents

## Abstract

*Leptospira* are bacteria that cause leptospirosis in both humans and animals. Human *Leptospira* infections in Uganda are suspected to arise from animal–human interactions. From a nationwide survey to determine *Leptospira* prevalence and circulating sequence types in Uganda, we tested 2030 livestock kidney samples, and 117 small mammals (rodents and shrews) using real-time PCR targeting the *lipL*32 gene. Pathogenic *Leptospira* species were detected in 45 livestock samples but not in the small mammals. The prevalence was 6.12% in sheep, 4.25% in cattle, 2.08% in goats, and 0.46% in pigs. Sequence typing revealed that *Leptospira borgpetersenii*, *Leptospira kirschneri*, and *Leptospira interrogans* are widespread across Uganda, with 13 novel sequence types identified. These findings enhance the East African MLST database and support the hypothesis that domesticated animals may be a source of human leptospirosis in Uganda, highlighting the need for increased awareness among those in close contact with livestock.

## Introduction

*Leptospira* is a genus of spirochete bacteria that includes pathogenic species responsible for causing leptospirosis in humans and animals. Leptospirosis is spread worldwide, with an estimated one million cases and 58900 deaths annually [1]. The genus *Leptospira* comprises approximately 64 genomospecies and over 250 serovars [2]. Although regional endemicity of certain *Leptospira* serovars and host-adapted types have been reported, small mammals, such as rodents and shrews are regarded as the main reservoirs in many instances [3]. Animal reservoirs do not show symptoms but are capable of shedding leptospires in urine for prolonged periods, consequently contaminating water and soil [4]. Infection in humans and domestic animals occurs through direct contact with mucosae or damaged skin with infected urine or abortive tissues or indirectly through contaminated water and soil [2,3].

In Uganda, there is growing evidence of *Leptospira* infection among febrile patients, and domesticated animals are speculated to be the source [5–7]. In one study, seroprevalence of 35% was estimated, with those involved in the skinning of cattle having 12 times higher odds of being seropositive [6]. Follow-up surveys of cattle, goats, sheep, and pigs across the country revealed *Leptospira* seroprevalence rates of 19.3%–27.8% [8–11]. Although this could mean endemicity and widespread *Leptospira* exposure among domestic animals in Uganda, the public health relevance of such exposures remains unresolved. Only animals with ongoing clinical infection or chronic carriers pose the risk of infection to humans and other animals or have the potential to contaminate the environment.

In Uganda, *Leptospira* infection based on real-time PCR assays has only been demonstrated in cattle, dogs, and pigs, with limited sequence typing data [12]. In the present study, we sampled livestock and small mammals at slaughter facilities across Uganda, to determine the status of *Leptospira* infection and circulating sequence types. Slaughter facilities offered convenient access to kidney specimens for PCR testing, enabling the detection of *Leptospira* in large livestock populations with wide geographical coverage. These facilities can also concentrate zoonotic agents and potentially spread infections to nearby communities through environmental contamination or by attracting disease reservoirs like small mammals [15].

## Materials and methods

### Research design

**Figure 1.**
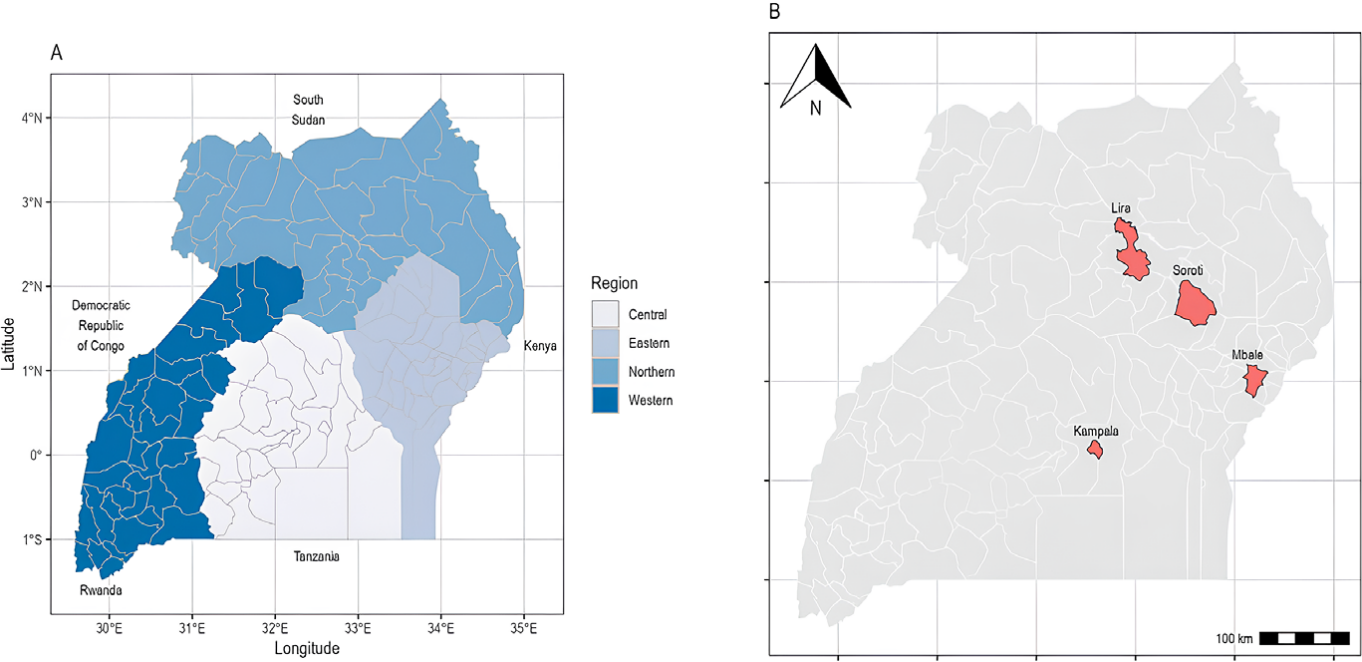
Map of Uganda showing the regions (**a**) and districts selected as sites for this cross-sectional study (**b**). Source of shapefiles: Uganda – Subnational Administrative Boundaries – Humanitarian Data Exchange (humdata.org) and World Administrative Boundaries – Countries and Territories – Opendatasoft.

Between December 2021 and October 2022, we conducted a cross-sectional study in selected livestock slaughter facilities across three of the four geographical regions of Uganda (East, North, and Central). In each region, the district with the largest number of daily slaughters for all species was selected as the study site, except in the East, where no one district slaughtered the highest number of all the livestock species. Instead, two study sites were recruited. The selected study sites were Lira in the North, Kampala in the Central, and Mbale and Soroti in the East (Figure 1). No site was recruited in the Western region following notification by key informants that a significant proportion of the livestock slaughtered in Kampala (our study site in Central) came from the West, and previous studies in slaughter facilities in Kampala have reported similar findings [15, 16].

### Sample size

Sample sizes were calculated using Epitools-epidemiological calculators [16], to estimate the overall true prevalence of *Leptospira* in Uganda without aiming to compare differences between the regions. The minimum sample estimates were 316 cattle (based on 7.2% prevalence in Ugandan slaughter cattle [13]), 53 each for goats and sheep (based on 1.2% prevalence reported in Tanzania [17]), 114 for pigs (assuming a conservative prevalence of 5%), and 99 for small mammals (based on a 3.5% prevalence from an unpublished survey conducted by the first author and colleagues at a wildlife-human interface in southwestern Uganda in 2016). The estimates considered a *lipL*32 real-time PCR with a sensitivity of 93% and specificity of 98.3% [18] and an error margin of 5%. However, as many samples as could be tested for each species were considered since these samples had already been collected to match the sample sizes for estimating *Leptospira* seroprevalence in the same population. *Leptospira* prevalence data for goats are based on reports from countries neighbouring Uganda due to missing local reports at the time the study was designed.

### Sampling of livestock

At each site, collection of samples from cattle and small ruminants (goats and sheep) was alternated daily over a 30 day-period to minimize the overrepresentation of animals with the same population characteristics. Pigs were sampled for 16 consecutive days, except in the Eastern region, where sampling was only possible for 10 days due to Easter festivals. An extra pig slaughter facility was enrolled in the East to compensate for this difference in sampling time. Consecutive collection of pig samples was considered because the daily slaughter stock turnover ranged between 80% and 100% in all sites at the time.

On each collection day, slaughtered animals were sampled opportunistically (the next animal was selected when the previous animal was completely sampled). From every animal chosen, a random piece of kidney that included the cortex and medulla and weighed at least five grams was collected aseptically into a sterile screw cap container. The sample volume was required for the tissue homogenization methods used in this study. Age (young, and adult), sex (male, and female), and breed (local, exotic, or cross) were noted for each animal, and information on the district of origin was obtained from consultation with the traders or animal movement permits held at the slaughter facilities. Samples were loaded in an ice-cooled box and dispatched daily to the Central Diagnostic Laboratory at the College of Veterinary Medicine, Animal Resources and Biosecurity (COVAB), Makerere University, Uganda. Samples arrived at COVAB on the same day except during collections from the Eastern and Northern regions, where arrival was the next day.

### Sampling small mammals

Small mammals were trapped at the same slaughter facilities where livestock was sampled, and in consenting homesteads within a 500-meter radius from the slaughter facilities. The number of homesteads enrolled per region was aimed at a cumulative trap effort of 200 trap nights, except in the central region where the effort was doubled because of the reported scarcity of rodents. For each homestead, two to five small Sherman traps (HB Sherman Traps, Tallahassee, USA) were set in houses, stores, kitchens, poultry houses, or surrounding vegetation. The traps were baited with a combination of ground nuts, peanut butter, sweet bananas, tomatoes, and silver cyprinid, depending on what was commonly reported as being gnawed by small mammals in each homestead. Trapping was done overnight, with the baits and successful traps replaced each morning. Captured animals were euthanized using diethyl ether and transported in ice-cooled boxes to the Central Diagnostic Laboratory, COVAB, Makerere University, where species identification was performed by an experienced zoologist based on phenotypic characterization and measurements of morphometric features [19]. The determination of sex and approximate age were based on external sexual characteristics. This was followed by dissection and extraction of the kidney, spleen, and part of the liver.

### Preparation of tissue homogenates and DNA extraction

Three grams of livestock kidney tissue was homogenized and reconstituted in 6 mL of sterile phosphate-buffered saline (pH 7.4; Rankem–RFCL, India). For the small mammals, 50% homogenate was prepared from a pool of both kidneys, the spleen, and part of the liver. Homogenization was achieved by crushing the tissues in stomacher bags (BA6040, Stomacher® 80, Seward Ltd., UK) using a ceramic pestle. DNA was extracted from 100 μl of tissue homogenate using the QIAamp DNA Mini Kit for blood or tissue (Qiagen, Hilden, Germany) according to the manufacturer’s guidelines. A dry spin was applied, and the DNA was eluted in buffer AE in two successive steps of 50 μl each and stored at -20 °C. For every extraction run, a *Leptospira*-positive homogenate was included as a positive extraction control, and pyrogen-free water was used as a negative extraction control.

### Isolation of Leptospira species

Kidney homogenates from 25% of the livestock samples and all the small mammals collected each day were cultured to isolate leptospires. Three 10-fold serial dilutions of each homogenate were made in 5 mL of commercial formulations of Ellinghausen-McCullough-Johnson-Harris (EMJH) medium in which supplements of albumin, polysorbate 80 and additional growth factors have been added (BD Difco™ *Leptospira* Enrichment EMJH, product 279,510, USA). The primary inoculates (dilution of 1/10) were discarded, and the two subsequent dilutions were incubated at 29.5°C for 2 days before checking for any signs of turbidity. Subsequent subcultures with visible turbidity were then made in 5 mL of fresh EMJH in which 5′-fluorouracil had been added at a concentration of 200 mg/L and examined every 7–14 days under a dark field microscope for visible leptospires. Cultures in which no visible turbidity or leptospires were observable after 14 weeks were autoclaved and discarded. DNA was isolated from suspected cultures, and the presence of pathogenic leptospires was tested using real-time PCR, as described below.

### Real-time polymerase chain reaction (PCR)

A TaqMan PCR assay targeting the *lipL*32 gene was used to detect pathogenic *Leptospira* in the DNA from livestock and rodent samples. The primers and probes used in this study were described previously by Villumsen et al. [20] and synthesized by Eurofins Genomics, France. The presence/absence of the bacteria was determined on a Quantistudio™ 5 PCR System (Applied Biosystems, Foster City, CA, USA) under the following conditions: pre- and post-cycling at 60°C for 30 s, holding at 50°C for 2 min, 95°C for 10 min, and 45 cycles of 95°C for 15 s and 60°C for 1 min. The final concentrations of the mixture in a reaction volume of 20 μl were: 1x TaqMan™ Fast Advanced Master Mix, 0.5x TaqMan® Exogenous Internal Positive Control mix (IPC), 0.5x IPC template (Applied Biosystems, Foster City, CA, USA), 1 μM each primer, 80 nM probe and 2.0 μl of DNA template. DNA from *Leptospira interrogans* serovar Icterohaemorrhagiae (strain RGA) and from a positive extraction sample were included as amplification controls, and 10X Block-Exp IPC® (Applied Biosystems, Foster City, CA, USA) and pyrogen-free water were used as negative amplification controls. A positive sample was defined as one that showed an exponential amplification curve in fewer than 41 cycles, with the fluorescence threshold set at 0.06.

### Identification of infecting Leptospira species

*Leptospira*-positive samples with cycle threshold (Ct) ≤36 cycles were typed using nested single-locus sequence typing (SLST) of the *secY* gene as described previously [21], and sequences of 245 bp fragments were searched against the BLASTn database for species identification (https://blast.ncbi.nlm.nih.gov/Blast.cgi). Multilocus sequence typing (MLST) was performed on the *secY*-positive samples using Scheme 1, which targets seven housekeeping genes, namely, *glmU*, *pntA*, *sucA*, *tpiA*, *pfkB*, *mreA*, and *caiB* [22]. The sequences were submitted to the PubMLST *Leptospira* database (http://pubmlst.org/leptospira, accessed in November 2023) to determine the allele and allelic profiles for sequence type identification. The sequences were analyzed using Bionumerics software 7.6.3 (Applied Maths, Belgium). *SecY* sequences and concatenated sequences from the MLST were imported to R 4.1.1 [23] using the Biostrings and msa packages, where multiple sequence alignments were generated using the clustal omega method, and distance matrixes were computed. Phylogenetic trees were constructed using the neighbour-joining method.

### Data analysis

The data were entered into Microsoft Excel® and analyzed in R version 4.1.1 [23]. Descriptive analysis of population demographics by animal species, breed, age, sex, and region of origin was performed, and the true *Leptospira* prevalence was calculated using the *epi.prev* function of the *EpiR* package, based on the Rogan-Gladen estimator. The input sensitivity and specificity of the PCR were 86% and 100%, respectively [20], with the method set to ‘blaker’.

### Ethical considerations

This study was approved by the Institutional Animal Care and Use Committees of the International Livestock Research Institute (Approval Number ILRI-IACUC2022–17), the School of Biosecurity, Biotechnical and Laboratory Sciences, College of Veterinary Medicine, Animal Resources and Biosecurity (COVAB), Makerere University (Approval number SBLS/HDRC/20/012) and the Uganda National Council for Science and Technology (Approval Number HS1563ES).

## Results

### Population characteristics of the sampled livestock and small mammals

Of the 2030 livestock sampled, 820 cattle, 335 goats, 114 sheep, and 761 pigs were included. Up to 78.47% (n = 1593) of the animals were adults. There were more female animals sampled, except for cattle, where 57.56% (472/820) were males (Table 1). Cattle, goats, and sheep were predominantly local breeds, while 65.70% (500/761) of the pigs were crossbred. The origin of 3.94% (n = 80) of the animals sampled could not be determined due to a lack of access to accompanying documentation.

**Table 1.** Population characteristics of the livestock (n = 2030) sampled during a cross-sectional study in slaughter facilities in Uganda

		Number of sampled animals (%)				
Category	Levels	Cattle	Goats	Sheep	Pigs	Total
Sex	Female	348 (42.44)	178 (53.13)	63 (55.26)	444 (58.34)	1,033 (50.89)
	Male	472 (57.56)	157 (46.87)	51 (44.74)	317 (41.66)	997 (49.11)
Age	Adult^a^	737 (89.88)	289 (86.27)	102 (89.47)	465 (61.10)	1,593 (78.47)
	Juvenile	83 (10.12)	46 (13.73)	12 (10.53)	296 (38.90)	437 (21.53)
Breed	Cross	130 (15.85)	61 (18.21)	2 (1.75)	500 (65.70)	693 (34.14)
	Exotic	0 (0.00)	0 (0.00)	0 (0.00)	2 (0.26)	2 (0.01)
	Local	690 (84.15)	274 (81.79)	112 (98.25)	259 (34.03)	1,335 (65.76)
Region of origin	Central	153 (18.66)	28 (8.35)	7 (6.14)	421 (55.32)	609 (30.00)
	Eastern	107 (13.05)	39 (11.64)	5 (4.38)	119 (15.64)	270 (13.30)
	Northern	454 (55.37)	191 (57.01)	70 (61.40)	210 (27.59)	925 (45.57)
	Western	43 (5.24)	65 (19.40)	25 (21.93)	1 (0.13)	134 (6.60)
	Across Tanzania border	12 (1.46)	0 (0.00)	0 (0.00)	0 (0.00)	12 (0.59)
	Undetermined	51 (6.22)	12 (3.58)	7 (6.14)	10 (1.31)	80 (3.94)
Total		820 (100)	335 (100)	114 (100)	761 (100)	2030 (100)

a Adult cattle were defined as ≥ 1.5 years, a goat as one ≥ 7 months, and a pig ≥ 6 months.

With a total of 877 trap nights, 117 small mammals were captured from the three regions, yielding an overall trap success rate of 13.34%. Most of the captures were from the Eastern (40.17%, n = 47) and Northern regions (37.61%, n = 44). Despite doubling the trapping effort in the Central region, only 26 small mammals were captured (4.81% success with 457 trap nights). There were more male (70.09%, n = 82) and adult (92.31%, n = 108) small mammals captured. The house rat (*Rattus rattus*) was the most common (65.81%, n = 77). The African pygmy mouse (*Mus minutoides;* 18.80%, n = 22), the house mouse (*Mus musculus;* 4.27%, n = 5), the African grass rat (*Arvicanthis niloticus*; 2.56%, n = 3), and the African giant shrew (*Crocidura olivieri;* 8.55%, n = 10) were also captured.

### Prevalence of Leptospira infection in livestock and small mammals based on the lipL32 PCR

*Leptospira* infection was detected in 45 of the 2030 livestock samples by PCR. Most of the infected livestock were adult (91.1%, 41/45), or from the Northern region (57.8%, 26/45) (Table 2). The estimated true prevalence of infection was highest in sheep (6.12%; 95% CI = 2.69–12.89), followed by cattle (4.25%; 95% CI = 2.91–5.98), goats (2.08%; CI = 0.91–4.38), and pigs (0.46%; CI = 0.12–1.31). Further statistical analysis of the association between *Leptospira* infection and age, sex, or region of origin was not performed due to the low number of positives observed (Table 2). None of the 117 small mammals were infected (0%; CI = 0.00–3.55). Culturing yielded four pre-sumptive *Leptospira* isolates from two cattle, one goat, and one house rat. However, the *lipL*32 PCR analysis of DNA from these isolates was negative, implying that they may have been non-pathogenic *Leptospira* species, and thus were not followed further.

**Table 2.** The proportion of *Leptospira*-infected livestock by species, sex, breed, age, and region of origin

		Number of positive samples (%)				
Category	Levels	Cattle	Goats	Sheep	Pigs	Total
Sex	Female	5 (1.44)	6 (3.37)	6 (9.52)	3 (0.66)	20 (1.94)
	Male	25 (5.30)	0 (0.00)	0 (0.00)	0 (0.00)	25 (2.51)
Age	Adult^a^	27 (3.66)	6 (2.08)	6 (5.88)	2 (0.43)	41 (2.57)
	Juvenile	3 (3.61)	0 (0.00)	0 (10.53)	1 (0.33)	4 (0.92)
Breed	Cross	4 (3.08)	0 (0.00)	0 (0.00)	2 (0.4)	6 (0.87)
	Exotic	0 (0.00)	0 (0.00)	0 (0.00)	0 (0.00)	0 (0.00)
	Local	26 (3.77)	6 (2.19)	6 (5.36)	1 (0.39)	39 (2.92)
Region of origin	Central	10 (6.54)	0 (0.00)	0 (0.00)	1 (0.24)	11 (1.81)
	Eastern	4 (3.74)	0 (0.00)	0 (0.00)	0 (0.00)	4 (1.48)
	Northern	14 (3.08)	5 (2.11)	5 (7.14)	2 (0.95)	26 (2.81)
	Western	1 (2.34)	1 (1.53)	0 (0.00)	0 (0.13)	2 (1.49)
	Across Tanzania border	1 (8.33)	0 (0.00)	0 (0.00)	0 (0.00)	1 (1.46)
	Undetermined	0 (0.00)	0 (0.00)	1 (14.29)	0 (0.00)	1 (1.25)
Total		30 (3.66)	6 (1.79)	6 (5.26)	3 (0.39)	45 (2.22)

a *Adult cattle were defined as ≥ 1.5 years, a goat as one ≥ 7 months, and a pig ≥ 6 months.*

### Leptospira species and sequence types

**Figure 2.**
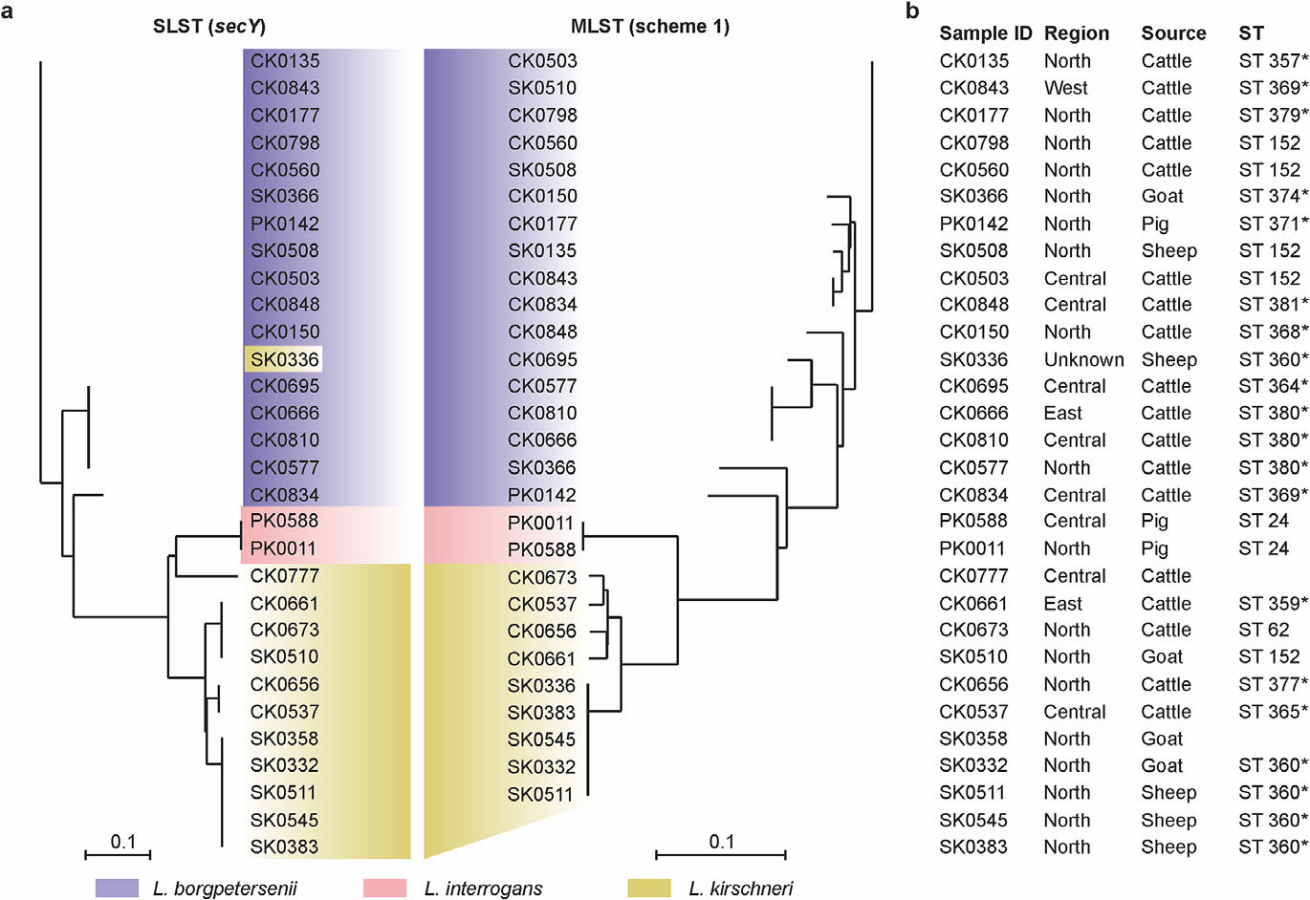
The phylogenetic relationship of leptospires detected in various slaughter animals by (**a**) single locus and multilocus sequence typing, with the region, source of samples, and the sequence types (ST) identified. (**b**). MLST alignment utilized concatenated sequences of the seven scheme 1 gene, and novel sequence types are denoted by an asterisk (*). The sequence type could not be identified for SK0358 and CK0777 due to failure in the amplification of the *caiB* and *tpiA* genes, respectively.

Of the 45 *Leptospira lipL32*-positive samples, 31 had a Ct ≤36 cycles. *SecY* typing was successful in 30 (96.77%) of the samples. *Leptospira borgpetersenii* was the most prevalent *Leptospira* species (n = 16) and was mostly found in cattle (n = 13), with goats, pigs, and sheep each having a positive sample. *Leptospira kirschneri* was detected in 5 cattle, 3 goats, and 4 sheep, and *Leptospira interrogans* were detected in 2 pig samples. MLST revealed 16 different sequence types (STs) in 29 of the 30 secY-positive samples, with ST152 and ST360 being the most prevalent and being detected in five animals each. ST 380 was detected in three animals, and ST 369 and ST 24 in two animals each. ST 62, ST 357, ST 359, ST 364, ST 365, ST 368, ST 371, ST 374, ST 377, ST 379, and ST 381 were found in one animal each. Several single-nucleotide polymorphisms were observed in the genes sequenced via MLST, leading to the identification of new alleles for these housekeeping genes and, consequently, 13 novel STs that were registered in the PubMLST database (Figure 2). These comprise ST 357, ST 359, ST 360, ST 364, ST 365, ST 368, ST 369, ST 371, ST 374, ST 377, ST 379, ST 380, and ST 381.

## Discussion

We detected infection with pathogenic *Leptospira* of the species *Leptospira borgpetersenii*, *Leptospira kirschneri* and *Leptospira interrogans* among apparently healthy cattle, goats, sheep, and pigs, suggesting their role as *Leptospira* carriers in Uganda. This finding has important implications for public health and animal health, as it highlights the potential risk of transmission through contact with these animals. *Leptospira* infection in livestock results in reproduction and production losses, such as milk yield reduction, stunting, abortions, and deaths. This could have far-reaching economic effects since cattle, goats, sheep, and pigs are the most common livestock kept in Uganda, and are a source of livelihood for up to 70% of households [24]. Infected livestock may also carry and shed leptospires in urine for weeks to years, consequently contaminating soil and water sources, and posing the risk of infection for humans [4].

From a systematic review of leptospirosis in Africa, livestock particularly cattle appear to be important hosts of several *Leptospira* serogroups, though few data are available to allow comparison of *Leptospira* infection in linked human and animal populations [25]. In East Africa, *Leptospira* exposures have been reported among febrile patients, slaughterhouse workers, and sugarcane plantation workers [26–28]. In Uganda, human *Leptospira* exposures have earlier been speculated to result from animal contact [5–7]. Findings from the current study indirectly build onto this speculation, especially that *Leptospira* sequence types identified in the current study belong to *L. borgpetersenii*, *L. kirschneri*, and *L. interrogans*, the same *Leptospira* species previously reported in febrile patients in Uganda [6], and elsewhere in East Africa [6,26,29,30].

The MLST results revealed the circulation of the same *Leptospira* sequence types within livestock species from different regions of Uganda, implying widespread *Leptospira* infection. This could be explained by animal movements and trade across regions within Uganda [31] and the neighbouring countries [32]. Twelve cattle sampled in our study were reportedly sourced from across the Tanzanian border, and one was *Leptospira* positive but did not qualify for sequencing (had a Ct of 38). *Leptospira* sequence type (ST) 152, one of the most detected STs in our study, was also detected in isolates from cattle in Tanzania [17]. Furthermore, the sharing of ST 152 between goats and cattle in the current study may imply interspecies transmission or a common source of infection, since cattle and goats are usually kept together in Uganda [33]. The identification of several other new STs within *L. borgpetersenii* and *L. kirschneri* in the current study may mean that the *Leptospira* strains circulating in Uganda are both novel and genetically diverse. While we also intended to characterize the local strains further by next-generation sequencing, we failed at isolating pathogenic leptospires in the present study. Future studies should consider isolation from clinical cases; target multiple sample types, including urine, blood, or kidney tissue; and employ a pre-screening test, such as PCR.

Comparable levels of *Leptospira* infection as found in livestock in the current study, have been reported elsewhere in East Africa. For example, a cross-sectional study of livestock sampled from slaughterhouses in Tanzania [17], reported pathogenic *Leptospira* infection was detected in 7.1% of cattle (n = 452), 1.2% of goats (n = 167), and 1.1% of sheep (n = 89). Earlier studies in Uganda revealed a *Leptospira* prevalence of 8.8% (n = 500) in slaughtered cattle [13], and 10.5% (n = 649) in slaughter pigs [14], compared to 4.3%; and 0.5% respectively reported in the current study. This could be because the other studies employed a more comprehensive sampling approach, which included kidneys, urine, and reproductive tissue, despite being based in slaughter facilities from only one region of Uganda and studying one livestock species each. In the current study, *Leptospira* prevalence in pigs was still comparably lower than in the other livestock species possibly due to the limited exposure risk associated with the semi-intensive systems under which most pigs in Uganda are kept. Further statistical analysis of the association between *Leptospira* infection and factors such as region of origin, age, and sex was not performed due to the low number of positive samples detected.

The absence of PCR-positive results in the 117 rodents or shrews captured near slaughter facilities in Uganda suggests that small mammals have a limited role in the community spread of *Leptospira.* This could also indicate that slaughter facilities in Uganda do not significantly contribute to *Leptospira* concentration. However, these conclusions may be undermined by the fact that slaughtered livestock originate from various locations and spend minimal time at these facilities. The predominance of the *Rattus rattus* species, known for staying close to human settlements with minimal habitat sharing with other rodents, may also have influenced the findings. The prevalence of *Leptospira* infection among *R. rattus* species is generally low even in environments where a high *Leptospira* prevalence is reported [34, 35].

Despite reports of *Leptospira* infection in rodents in some parts of Africa [36, 37], their role as *Leptospira* reservoirs in East Africa seems limited. A two-year cross-sectional survey conducted at 12 randomly selected sites in Tanzania revealed no *Leptospira* infection in any of the 384 rodents captured [17]. The first author of the present study has earlier participated in two independent captures of small mammals conducted in a rural agricultural environment and at a wildlife-human interface in Uganda and found *Leptospira* infection in 2.6% (n = 234), and 3.5% (n = 198) respectively (unpublished). Despite this, small mammals or wildlife reservoirs may still contaminate environmental sources such as water, and soil in grazing fields from which domesticated animals are indirectly infected. Given their close interaction with humans and larger urine volumes, livestock are likely the more important carriers and sources of human *Leptospira* infection in Uganda, compared to small mammals.

Our study documents the livestock reservoirs of pathogenic leptospires in Uganda and the circulating *Leptospira* species and sequence types among these reservoirs, with the long-term goal of informing prevention and control measures for leptospirosis in Uganda. The *Leptospira* sequence types identified in the present study, including the novel ones, contribute to the MLST database for East Africa and offer a basis for further research to isolate and identify the serogroups and serovars to which these novel sequence types could belong. Our findings also build onto the existing hypothesis that domesticated animals could be a source of human *Leptospira* infection in Uganda, emphasizing the importance of raising awareness among individuals in regular contact with livestock, such as farmers, slaughterhouse workers, and veterinarians.

## Data Availability

All necessary data have been presented in the manuscript, and further specific requests can be through the corresponding authors.
